# The Muriated Tincture of Iron as a Styptic

**Published:** 1872-10

**Authors:** W. F. Westmoreland

**Affiliations:** Professor of Surgery in the Atlanta Medical College


### MEDICAL AND SURGICAL JOURNAL.

VOL. X.—OCTOBER, 1872.—NO. 7.

---

### ORIGINAL COMMUNICATIONS.

---

### MURIATED TINCTURE OF IRON AS A STYPTIC:

### *WITH CASES ILLUSTRATING SOME OF ITS ADVANTAGES.*

BY W. F. WESTMORELAND, M.D.,

*Professor of Surgery in the Atlanta Medical College.*

[Presented to the Georgia Medical Association, at its Annual Meeting in 1872.]

Since the experiments of MM. Pravaz and Lallemand, nearly a quarter of a century ago, in which they demonstrated the possibility of coagulating the blood within an artery or vein by the injection of the perchloride of iron, and thus obliterating the vessel, the preparations of iron have occupied a prominent place as styptics. The preparations, however, most in favor, and which have been almost exclusively relied upon by the profession, are the perchloride and the subsulphate; and in the majority of instances, they have been used with the idea of arresting the hemorrhage by coagulating the blood at the mouth of the bleeding vessel, without any reference whatever to the more important action of contracting the tissues composing the walls of the tube, thus reducing the calibre of the bleeding vessel, and staying the hemorrhage.

In 1863 and '64, it was my fortune to be in charge of a large military hospital in Atlanta, Georgia, in which were received the wounded of the Western army, then operating in Northern Georgia and Tennessee. After several of the prominent battles, secondary hemorrhage from stumps, or wherever an artery was ligated, was unusually and alarmingly frequent.

After the battle of Murfreesboro, I lost, in one or two weeks, twelve or fifteen cases from secondary hemorrhage, and often after ligating the bleeding vessel more than once. In one case, I ligated the vessel four times—the first ligation being at the junction of the middle and lower third of the femoral artery, and the fourth and last at the external iliac. From the eighth to the fifteenth day after each operation, the artery gave way at the point ligated, with a frightful flow of blood. This was the usual course in such cases; in the majority, however, the wounded man would die from loss of blood after the first or second ligation.

I do not propose, in this paper, to discuss this peculiar condition of the arteries, or the causes producing it, but to call the attention of the members of the Association to a mode of arresting hemorrhage that I adopted in this trying ordeal, and one which I found almost invariably successful, even where the larger arteries were involved.

After using the various ligatures, as silk, animal, and silver wire, with the terrible results above alluded to, I determined to adopt some other plan, and commenced experimenting with styptics, compression, etc.

Owing to the blockade then existing in the Confederacy, many of the leading articles in medicine could not be procured, or at least in sufficient quantities to supply the demands of the sick and wounded. The perchloride and subsulphate of iron, in which I then had more confidence than all the styptics in the arrest of hemorrhage, could not be procured in sufficient quantities to accomplish much—one or two cases usually exhausting a month's supply. In such an emergency, I commenced the use of the muriated tincture of iron.

In hemorrhage from the smaller arteries, I found that I could readily arrest the flow by coagulating the blood with the muriated tincture of iron, but usually the coagulation was not so prompt or effectual as with the perchloride or subsulphate. I found, also, that if I relied alone on the clot, whether produced by the muriated tincture or the subsulphate, that the clot thus formed would, in from two to four days, soften, or in some other way become detached, and, if the artery was of much size, would be followed by a recurrence of the hemorrhage. I found, however, that if I brought the muriated tincture of iron directly in contact with the bleeding vessel, and for a few minutes continued it, removing all clots as soon as formed, thus immersing the artery and surrounding tissues in this styptic, that if the artery was small, the hemorrhage would be arrested by the contraction alone of the coats and sheath of the artery, assisted, in some instances, by the contractions of the surrounding tissues. To produce this desirable result, it was found necessary to arrest the hemorrhage to the extent possible by compressing the artery, cleansing the wound of clots, pus, or other substances covering the bleeding vessel, placing the parts in a position favorable to the application of the styptic, and above all, to keep the muriated tincture of iron in contact with the wounded artery a sufficient length of time to insure the requisite contraction.

After thoroughly satisfying myself as to the effects of this preparation of iron by repeated applications to the smaller arteries, I determined, the first opportunity that offered, to try it upon the larger ones. I had to wait but a short time, as I had then in the hospital a lad seventeen or eighteen years old, from a Mississippi regiment, who had received a wound of the thigh, the ball passing in the vicinity of the femoral artery, perhaps wounding its sheath. In ten or fifteen days, both openings healed, and a few days later, the coats of the artery gave way, and in a few hours, an immense tumor formed in its vicinity. An incision was made over the tumor, the wounded artery reached, and a common silk ligature placed round both the distal and proximal ends of the wounded vessel. In ten or twelve days, the vessel gave way at the point of ligation of the proximal extremity, with a copious flow of blood. A silver ligature was then applied two or three inches above the first, which, in ten or twelve days, was followed by the same unpleasant results, and this time with a copious hemorrhage, the patient bleeding to syncope. Feeling that a further ligation of the vessel would be followed by the same result, and having determined to try my new mode of arresting secondary hemorrhage upon the larger arteries, I at once got ready to make the application.

I had an assistant to compress the artery over the ramus of the pubis, so as to completely arrest the hemorrhage. I then increased the size of the incision for the previous ligature, so as to bring the end of the severed artery completely in view. The wound was then filled with the muriated tincture of iron, thus immersing the coats of the artery, the sheath, and the surrounding tissue, in the styptic. With the finger, the position of the end of the artery was frequently changed, so as to bring every portion of the exposed vessel in contact with the iron. In five to ten minutes, there were some clots formed, and there was more or less hemorrhage from the granulations. The clots, with the iron, were all removed, the wound cleansed with a sponge, and the incision again filled with fresh tincture. In five or ten minutes, the wound was filled with charpie, saturated with the tincture of iron, and well packed. The edges of the wound were brought together over the charpie, and held in position by strips of adhesive plaster. The limb was placed in a comfortable position, and in thirty minutes after the dressing was commenced, the artery was turned loose. There was no hemorrhage. Forty-eight hours after the first dressing, the artery was again compressed by a reliable assistant, and the dressing removed; fresh lint, saturated as before with the tincture of iron, was again packed in the wound, and the edges brought together over it, as in the first dressing, with adhesive strips. This dressing was allowed to remain five or six days, at which time it was detached by pus, the lint was removed, and the wound filled with fresh charpie, this time without the iron. The artery in this dressing was compressed as before. In three or four weeks, the wound was healed, and in five weeks from the first application of the iron, the patient received a furlough, and left the hospital for his home in Mississippi.

In the report of this case, as in those which follow, I regret that I am not able to give names, regiments, dates, and other particulars; but as I lost my records during the war, I am forced to make the reports from memory, and consequently, can only give the prominent facts in each case; and, again, I propose only to mention such cases as will strikingly illustrate some of the advantages of this mode of arresting secondary hemorrhage.

A few weeks after the above case was discharged, a young soldier entered the hospital with an amputation at the shoulder-joint. A week or more after his entry, and ten or twelve days after the amputation, I was passing through the ward in which he was lying, and heard him, in great alarm, say to the nurse, that he was bleeding to death; and truly did it look so, for in less than half a minute, it had flooded his bunk, and before I reached him, had covered the floor near his bunk with blood. It happened that the stump was exposed, and had healed by the first intention, with the exception of a point half an inch wide, through which the ligature protruded. I grasped the lips of the wound, and, by compressing the points ununited, promptly arrested the flow, holding it in that position and compressing the stump until I could get an assistant with a key to compress the subclavian artery. The hemorrhage was, without the possibility of a doubt, from the severed axillary artery. After getting everything ready, and my assistant satisfied of the thorough compression of the subclavian, I turned loose the lips of the wound, and at once tore open the flaps so as to intro-my hand and remove the clots which had accumulated. I then, with a syringe and cold water, washed out the wound, cleansing it of all clots, pus, etc., and filled the stump (the flaps making a sort of pocket) with the muriated tincture of iron. I kept my fingers in the vicinity of the artery. The compression was not sufficiently perfect to prevent an occasional gush of blood, which made it necessary to change the iron in the stump several times, requiring half an hour or more before I was satisfied to introduce the charpie saturated with the styptic, as in the case of the femoral artery just reported. The stump was well packed with the lint, and the flaps brought together securely over it by means of strips of adhesive plaster, and all secured with a roller bandage around the body. The compression of the artery was kept up from three-quarters to an hour from the first application of the iron. In forty-eight hours, the subclavian artery was again compressed, the lint removed, and fresh lint, again saturated with the muriated tincture of iron, introduced, and the flaps, as in the first instance, secured by adhesive strips and a roller bandage. In a few days after, the stump was dressed again in the same way. The patient recovered rapidly, and without the least hemorrhage after the first dressing.

The following case is one of interest, as it could not be definitely ascertained what artery was wounded, and the mode of applying the styptic differs from the cases just reported:

A soldier, thirty-five or forty years of age, received a gun-shot wound through the upper portion of the thigh, and from its direction, it was impossible to say what artery was wounded, whether the femoral or profunda. He had suffered two copious hemorrhages, and after the third, more copious than the two preceding, and which was arrested by the compression of the artery as it passed under Poupart's ligament, I determined to arrest, if possible, the hemorrhage in this case by the injection of the styptic into the wound. After securing the artery by compression, with Davidson's syringe and water, I succeeded in thoroughly cleansing the track of the wound of all clots of blood, pus, or other fluids, and at once injected the muriated tincture of of iron by means of the same syringe. To insure the contact of the iron with the bleeding vessel, the tube of the syringe was introduced in one of the openings, and the other was closed, thus distending the parts with the styptic. The wound was kept distended for half hour or longer, when the limb was placed in a comfortable position, and the compression of the artery removed. There was no further hemorrhage, and recovery was rapid and perfectly satisfactory.

This case illustrates a class of cases which were troublesome, and which, after the appearance of hospital gangrene, were very frequent. I have, however, with one exception, invariably succeeded, in the way above indicated, in promptly arresting hemorrhages in the track of the ball in gun-shot wounds, whether the result of the original injury or from the ravages of hospital gangrene. I should state, however, that where satisfied that the hemorrhage is from the principal artery, as the femoral or brachial, I would not rely upon the injection of the styptic, but would, by incision, bring the artery to view, and thus insure a more satisfactory application, and at the same time make the amount of compression by means of charpie, which, in the majority of cases in the large arteries, is necessary for the prompt arrest of the hemorrhage.

The next case that I shall mention in this connection was in a patient who had suffered for several weeks with typhoid fever, followed as is not unusual, by an abscess of the parotid gland. I was called to the case by the attending physician, to ligate the carotid artery. Upon my arrival, I found that the patient had had three copious hemorrhages from the cavity of the abscess, with an interval of three or four days between each flow of blood. The last was more copious than the two former, the patient having bled to syncope. I found his bedding, as well as his clothing, flooded with blood, and an immense clot filling the cavity of the abscess. It was evident, from the rapid flow, that the hemorrhage was from an artery of considerable size, but from what branch of the external carotid, it was impossible to say. I suggested, that instead of ligating the artery, we should try the muriated tincture of iron, which was readily agreed to by Dr. McKown, of Jonesboro, Georgia, who was then in attendance. After getting the iron and lint in readiness, and constructing a compress for the primitive carotid artery, notwithstanding the patient was greatly exhausted from the loss of blood a few hours previously, we at once commenced the application. After satisfying ourselves that the artery was sufficiently compressed, I freely laid open the walls of the abscess, so as to introduce my fingers and remove the clots. The abscess was now thoroughly cleansed with a syringe and cold water, and after removing the water with a sponge, the cavity was filled with the muriated tincture of iron, and by means of the finger introduced into the cavity, the iron was made to come in contact with every portion of the walls of the abscess. In from eight to ten minutes after its introduction, the tincture was removed with a piece of sponge, with all the clots, and the cavity again filled with the fresh tincture of iron. At the expiration of from five to ten minutes, the cavity was filled with lint saturated with the styptic, and the lips of the wound brought together and secured by adhesive strips. After removing the soiled bedding and clothing, and placing the patient in a comfortable position, the compression was removed from the artery. There was no further hemorrhage, and the patient recovered without an unpleasant symptom.

I could give numerous such cases as the above, occurring before and since the war, but feel that the above sufficiently illustrate the advantages of this styptic in the arrest of secondary hemorrhage.

I will mention one other case; in this, however, the application was not successful, the patient dying in a few days after the application, from loss of blood:

A soldier, twenty-five or thirty years of age, of good constitution, received a gunshot wound of the face during the battle of the 22d July, at Atlanta. The ball passed through the malar and superior maxillary of the left side, opening the antrum. Eight or ten days after his reception in the hospital, then at Milner, Georgia, he had a copious hemorrhage. The carotid upon that side was compressed, which arrested the hemorrhage; the wound was cleansed with a sponge and water, and the muriated tincture of iron injected in it, as in cases above mentioned. The hemorrhage ceased; three or four days later, however, while asleep at night, he had another hemorrhage, which was not discovered until he was completely exhausted from the loss of blood, and died in a few minutes after I reached his bed.

An examination after death revealed the fact that it was the internal maxillary artery wounded, and that the styptic did not come in contact with the bleeding vessel. The injection filled the antrum; but, from a clot in the track of the ball, which was not removed by the water, the iron, as above stated, did not not reach the bleeding vessel.

I would further state, that since the fall of 1863, soon after the battle of Chickamauga, I have, in every case where admissible, adopted the plan above suggested for the arrest of secondary hemorrhage, and have never failed in promptly and permanently arresting the hemorrhage in but the single instance above mentioned, where the post-mortem revealed the fact that the styptic did not come in contact with the bleeding vessel.

I would not be understood to advance the idea, that in the larger arteries this styptic, however thoroughly applied, would be of itself sufficient to arrest the flow of blood. I desire to make prominent the importance of compression in connection with this styptic at the mouth of the bleeding vessel, as without this precaution, we will often fail to arrest promptly the flow even in arteries of medium size. The amount of compression necessary, however, will depend, to a great extent, upon the size of the vessel, the cause producing the hemorrhage, and the age of the subject. The necessary compression can always be secured by exposing the vessel and filling the wound with lint—commencing by pledgets immediately over the wounded artery, and securing the lint in position by bringing the lips of the wound together by means of adhesive plaster. This I have demonstrated in more than one instance even in the largest vessels, as the femoral and axillary arteries.

After the close of the war, I determined to study the action of the tincture of the muriate of iron upon the blood vessels and tissues of the inferior animals, and, in 1866, made a series of experiments, which I have repeated before the different classes in attendance upon lectures in the Atlanta Medical College every year since, and invariably with the same results. These experiments were made upon the dog, and the tincture of the muriate of iron used was made in accordance with the formula of the United States Pharmacopœia, and was as nearly pure as I could conveniently procure.

I found that the first effect upon the tissues—as the muscular, cellular, and fibrous—when this styptic was brought in contact with them, was a marked contraction, they becoming more dense and unyielding; that this contraction would increase for the first fifteen or forty minutes, when the tissues would become comparatively hard and dense; that the younger the animal, the more prompt and marked was the contraction. I also noted that this contraction was not so manifest upon the same tissues in the dog as in the human subject. I found that when I dissected up the artery with the sheath intact, and made a section of the vessel with the sheath, that the hemorrhage was much more promptly arrested than from the same vessel when the sheath was removed, and the coats of the artery alone exposed to the action of the styptic. I was able to demonstrate that, in the smaller vessels, contraction of the sheath and the surrounding tissues by the tincture would suffice to arrest the hemorrhage, even where the iron did not come in contact with the vessels, and where there were no clots formed to account for the arrest of the flow. I also found that, in arteries of medium size, it was impossible, in many cases, to permanently arrest the hemorrhage without compressing the artery so as to first arrest the flow—thus permitting the styptic to come in contact with the bleeding vessel and surrounding tissues; that if this precaution was not taken, the tincture of iron, when introduced, would form clots as it came in contact with the blood, which would be separated from the bleeding vessel by the constant flow, and thus prevent the action of the iron upon it; that if the hemorrhage was arrested in this way, even in arteries of medium size, it was by the formation of clots, and, in the majority of instances, just so soon as the clot softened, the hemorrhage would recommence. I further demonstrated the fact, that hemorrhage from the femoral artery of the dog could be completely and permanently arrested by the action of this styptic upon the walls of the vessel, even when the sheath was removed. I have repeatedly, in the young animal, dissected up the femoral artery for two or three inches, so as to leave only the tissues composing the walls of the artery, and when in this condition, passed a ligature around the lower portion of the artery exposed, so as to prevent the possibility of hemorrhage from the distal extremity, and then severed the artery above the ligature, thus having protruding from the wound an inch and a half or two inches of the denuded vessel. If, now, compression be made above, so as to completely arrest the flow, and the vessel immersed in the muriated tincture of iron for from ten to twenty-five minutes, the artery will contract to such an extent as to completely arrest the hemorrhage, even after all compression is removed. This last experiment was not *always* successful to the extent of completely arrresting the flow; but when the animal was young, and care was taken to remove any clots from the mouth or walls of the vessel, so that the styptic might have its full action upon the tissues, it rarely failed.

The artery, after such an application as the above, would present in a short time the appearance of a cord; but, strange to say, in the majority of such cases, I found that comparatively but a small portion of the artery thus acted on would be thrown off as an eschar; and when exposed in the same way, with the sheath intact, I have rarely seen a slough proper.

In other cases, where the hemorrhage was not completely arrested, lint was placed over the severed artery, and the wound closed with sutures. In examining the wound and arteries at different periods after such experiments, we usually found, on the second or third day, considerable inflammation; on the fourth to the sixth day, the wound would be cleansed by throwing off a very thin eschar, sometimes amounting to nothing more than the coagulated blood attached to the tissues—leaving, in the majority of cases, a healthy granulating surface. A fact, however, to be noted as one upon which the success of the application, to a great extent, depends, is, that the eschar from the walls or tip of the artery is not, as a rule, thrown off until some days later—say from eight to ten—than the eschar from the wound made in the soft parts.

After coming in contact with the numerous cases of secondary hemorrhage above alluded to, and with such marked success in controlling them, and satisfying myself in regard to the action of the styptic by the experiments upon the inferior animals, it occurred to me that aneurisms might be treated in the same way, without greater risk than by the ligature, and even much less when the aneurism was the result of a diseased condition of the artery. I determined to try it upon the first favorable case that presented. I did not have long to wait.

In 1867, I was called to see Mr. D., of Atlanta, Georgia, a man twenty-two years old, and of most excellent constitution, who, some months before, had received a gun-shot wound of the shoulder, the ball passing in the vicinity of the axillary artery. I found Mr. D. suffering from a pulsating tumor, the size of an orange, near the middle portion of the axillary artery, the result of the wound received a few months before. Active treatment was, by the patient, deferred for a time. When I was recalled, three or four weeks later, I found it perhaps three times the size it was my first visit—the tumor now resting under the inferior border of the pectoral muscle, the wall thin, pulsation distinct, and the patient suffering intensely.

I at once determined, if the patient would consent, under a fair representation of facts, to attempt its cure with the muriated tincture of iron—not, however, in the way suggested and practiced by M. Pravaz, by injecting a preparation of iron into the aneurismal sac, but to lay open freely the sac, and make the application directly to the wounded vessel. I presented to him a true statement of his case—the different modes adopted for the cure of such cases, the statistics, etc., in the ligation of the subclavian for aneurism. I then gave him the plan I intended to adopt if he consented—telling him frankly that, so far as I knew, it was untried, but gave him the result that I had met with in arresting hemerrhage by closing the artery by this plan. He promptly decided to try the new mode. Two days later, assisted by Drs. N. D'Alvigny, J. F. Alexander, J. M. Johnson, and others, I performed the following operation:

After placing the patient fully under chloroform, and satisfying myself of the thorough compression of the subclavian as it passed over the first rib, with one sweep of the bistoury, I laid open the aneurismal sac sufficiently to introduce my hand and remove the clots and blood contained in the tumor. After cleansing the sac to the extent possible—and at no time was it thoroughly cleansed, as the compression could not be made sufficiently perfect to completely arrest the hemorrhage—it was filled with the muriated tincture of iron, and, with the fingers introduced, the iron was brought in contact with the bleeding vessel. All clots were removed as soon as formed. Three or four times the sac was cleansed to the extent possible, and each time refilled with the fresh tincture. For thirty minutes the sac in this way was kept filled with the styptic, when, with pledgets of charpie saturated with the tincture, I commenced filling it, the first pledgets being placed directly over the wounded artery. After filling the sac well with charpie, the lips of the wound were brought together by adhesive strips, the soiled clothing and bedding were removed, the patient placed in a comfortable position, and then the compression was removed from the subclavian artery. No pulsations were perceptible in the radial artery. A striking feature in this case was the rapidity with which the sac was reduced when filled with the styptic; the incision in the skin, at the close of the operation, was at least one-third less than before the application of the iron.

For forty-eight or seventy-two hours, the patient had considerable fever. The dressing was not disturbed; one or two students were in constant attendance, day and night, with a compress in readiness to compress the artery, if necessary. Nothing unpleasant occurred until the night of the thirteenth day, when I received a message that the patient was bleeding copiously. I will not attempt to depict my feelings upon the reception of this message. Upon my arrival, I found the patient had lost only a few ounces of blood; but as the artery was being thoroughly compressed by an intelligent medical student, I had no other thought than that the hemorrhage was from the axillary artery. I at once commenced the removal of the lint, and soon found, to my surprise and joy, that the hemorrhage was from one of the muscular branches supplying the pectoralis major. I at once ceased the removal of the lint, and applied fresh lint, saturated with the muriated tincture of iron, to the bleeding point. Was this hemorrhage connected with, or produced by, the increase in the size of the artery, as the result of the attempt to establish the collateral circulation? Five days later, being the eighteenth day after the operation, I removed the lint from the wound, with the exception of the portion directly in contact with the vessel, which was not yet detached. On the twenty-third day, all the lint was removed, and the entire wound covered by healthy granulations. Nothing unpleasant occurred after this, and in due time the wound healed by the granulating process, and the patient entirely recovered.

It will perhaps be alleged by many that I was not justified in performing this operation, and from their stand-point, perhaps I was not; but from the numerous cases, the character of which has been given above, I had such confidence in this styptic, with compression, in controlling hemorrhage, that I did not hesitate for a moment to perform it.

In conclusion, gentlemen, permit me to state that I do not recommend this mode of arresting hemorrhage in recent injuries, as the ligature or torsion is certainly greatly to be preferred, where admissible. It is particularly in secondary hemorrhages, the result of some constitutional or local defect, or in the ulcerative or sloughing process, as in some forms of abscess and phagedenic degeneration of the tissues, that I recommend it. And in addition to the prompt arrest of hemorrhage under such circumstances, there are but few better applications to the diseased or relaxed tissue, as my experience has demonstrated the fact that, in the majority of such cases, the application of the styptic is followed by healthy granulations.

